# Supplementary material for: Imaging fascicular organization of rat sciatic nerves with fast neural electrical impedance tomography
Source: Nat Commun. 2020 Dec 7;11:6241. doi: 10.1038/s41467-020-20127-x (PMC7721735; doi:10.1038/s41467-020-20127-x)
Supplement: Supplementary file 3 — Reporting Summary [file 41467_2020_20127_MOESM3_ESM.pdf]

nature research

Corresponding author(s): Svetlana Mastitskaya

Last updated by author(s): Oct 28, 2020

Reporting Summary

Nature Research wishes to improve the reproducibility of the work that we publish. This form provides structure for consistency and transparency in reporting. For further information on Nature Research policies, see our Editorial Policies and the Editorial Policy Checklist.

Statistics

For all statistical analyses, confirm that the following items are present in the figure legend, table legend, main text, or Methods section.

n/a	Confirmed	
☐	☒	The exact sample size (*n*) for each experimental group/condition, given as a discrete number and unit of measurement
☐	☒	A statement on whether measurements were taken from distinct samples or whether the same sample was measured repeatedly
☐	☒	The statistical test(s) used AND whether they are one- or two-sided<br>*Only common tests should be described solely by name; describe more complex techniques in the Methods section.*
☐	☒	A description of all covariates tested
☐	☒	A description of any assumptions or corrections, such as tests of normality and adjustment for multiple comparisons
☐	☒	A full description of the statistical parameters including central tendency (e.g. means) or other basic estimates (e.g. regression coefficient) AND variation (e.g. standard deviation) or associated estimates of uncertainty (e.g. confidence intervals)
☐	☒	For null hypothesis testing, the test statistic (e.g. *F*, *t*, *r*) with confidence intervals, effect sizes, degrees of freedom and *P* value noted<br>*Give P values as exact values whenever suitable.*
☒	☐	For Bayesian analysis, information on the choice of priors and Markov chain Monte Carlo settings
☒	☐	For hierarchical and complex designs, identification of the appropriate level for tests and full reporting of outcomes
☒	☐	Estimates of effect sizes (e.g. Cohen's *d*, Pearson's *r*), indicating how they were calculated

Our web collection on statistics for biologists contains articles on many of the points above.

Software and code

Policy information about availability of computer code

Data collection	Custom code for operating the ScouseTom EIT recording system. Consists of:<br>1) Firmware code for the ArduinoDue microcontroller (https://www.arduino.cc/en/Main/Software)<br>2) MATLAB (Version r2018b, The MathWorks, Natick, USA).<br>ScouseTom hardware and software information is available at: https://github.com/EIT-team/ScouseTom.<br>All scripts and processing code used in the study are available at: https://github.com/EIT-team/.
Data analysis	EIT image reconstruction is performed with an open-source fast forward solver PEITS (https://github.com/EIT-team/PEITS) and a custom MATLAB code (Version r2018b, The MathWorks, Natick, USA, custom code available at: https://github.com/EIT-team/). Visual inspection of reconstructed images is performed with an open-source visualization application Paraview (Version 5.7.0, https://www.paraview.org/, Sandia National Labs, Kitware Inc, and Los Alamos National Labs, USA).<br>MicroCT image reconstruction is performed with Nikon CT Pro 3D (Version XT 4.4.4, Nikon Metrology, Tring, UK). Visual inspection of reconstructed microCT scans is performed with Seg3D (Version Seg3D2-2.4.2, http://www.seg3d.org, NIH Centre for Integrative Biomedical Computing, SCI Institute, University of Utah, USA), and reconstructed microCT scan images in XY plane are analyzed in ImageJ (Version 1.52p, National Institutes of Health, USA).<br>Analysis of neural tracer histology images is performed in ImageJ (Version 1.52p, National Institutes of Health, USA).<br>Data analysis is performed with MATLAB (Version r2018b, The MathWorks, Natick, USA).

For manuscripts utilizing custom algorithms or software that are central to the research but not yet described in published literature, software must be made available to editors and reviewers. We strongly encourage code deposition in a community repository (e.g. GitHub). See the Nature Research guidelines for submitting code & software for further information.

Data

Policy information about availability of data

All manuscripts must include a data availability statement. This statement should provide the following information, where applicable:

- Accession codes, unique identifiers, or web links for publicly available datasets
- A list of figures that have associated raw data
- A description of any restrictions on data availability

The data that support the findings of this study are available within the Article and Supplementary Files, or available from the corresponding author upon request. The source data underlying Fig. 2 and Fig. 5 are provided as a Source Data file.

Field-specific reporting

Please select the one below that is the best fit for your research. If you are not sure, read the appropriate sections before making your selection.

☒ Life sciences ☐ Behavioural & social sciences ☐ Ecological, evolutionary & environmental sciences

For a reference copy of the document with all sections, see nature.com/documents/nr-reporting-summary-flat.pdf

Life sciences study design

All studies must disclose on these points even when the disclosure is negative.

Sample size	From our experience in collecting physiological data, we can reliably localize the source of electrical activity within the nerve with n=5 per group if differences between the centres of mass of the imaged fascicles are as large as 2.25 standard deviations of the radial and angular position of each individual fascicle, which is likely to be a physiologically significant difference. The significance level was set at 0.05 and the power of the test was set at 0.8, for three groups of fascicles.
Data exclusions	Exclusion criteria are described in the main text of the paper. Out of eight sciatic nerves in four animals in the study, three nerves were excluded for the reasons not associated with the main method (EIT) described in the paper. In three nerves that were excluded, the neural tracing failed in one or two fascicles and/or the mechanical damage to the nerve during tracer injection precluded subsequent successful detection of the CAP:<br>Animal 1 - left sciatic nerve is excluded due to mechanical damage during surgery, which led to weak CAPs and weak dZ, which resulted in failure of EIT recording.<br>Animal 2 - both sciatic nerves are included in the study.<br>Animal 3 - during EIT recordings on the left sciatic nerve, there was significant electromagnetic noise/interference caused by equipment operated in the neighboring laboratory. The source of the noise was identified too late, and the nerve had to be excluded from the study.<br>Animal 4 - neural tracing failed in the left sciatic nerve due to operator's mistake during injection of the tracers. The nerve was excluded from the study because the failure of the tracing precluded cross-validation between techniques in this nerve.<br>Criteria for exclusion of collected impedance traces from subsequent reconstruction process were as follows: 1) Injection/measurement on faulty electrode (characterized by extremely high noise compared to the rest of the electrodes); 2) DC saturation of raw signal; 3) deltaV background noise > 3 microvolt.
Replication	Impedance changes in four out of five nerves included in the study were taken in two repeats for each individual fascicle. Only in animal 1 (right sciatic nerve) we didn't have enough time to collect EIT data in repeats as this was the very first experiment. We have spent too much time trying to collect EIT data on the left sciatic nerve which appeared to be damaged. At least two repeats were taken in all other nerves, all resulted in successful EIT recordings.
Randomization	Randomization is not relevant to this study. This is a method development study for imaging the internal structure of the peripheral nerves. The evoked fascicular activity was detected with the Electrical Impedance Tomography, and the resulting reconstructed images of activity were validated against gold-standard techniques.
Blinding	Blinding was not relevant to this study. This is a method development study for imaging the internal structure of the peripheral nerves. The evoked fascicular activity was detected with the Electrical Impedance Tomography, and the resulting reconstructed images of activity were validated against gold-standard techniques.

Reporting for specific materials, systems and methods

We require information from authors about some types of materials, experimental systems and methods used in many studies. Here, indicate whether each material, system or method listed is relevant to your study. If you are not sure if a list item applies to your research, read the appropriate section before selecting a response.

Materials & experimental systems

n/a	Involved in the study
☒	☐ Antibodies
☒	☐ Eukaryotic cell lines
☒	☐ Palaeontology and archaeology
☐	☒ Animals and other organisms
☒	☐ Human research participants
☒	☐ Clinical data
☒	☐ Dual use research of concern

Methods

n/a	Involved in the study
☒	☐ ChIP-seq
☒	☐ Flow cytometry
☒	☐ MRI-based neuroimaging

Animals and other organisms

Policy information about studies involving animals; ARRIVE guidelines recommended for reporting animal research

Laboratory animals	Adult male Sprague-Dawley rats (400-450 g, or 4-5 months old) were used in this study.
Wild animals	The study did not involve wild animals.
Field-collected samples	The study did not involve samples collected from the field.
Ethics oversight	All experiments were performed in accordance with the European Commission Directive 2010/63/EU (European Convention for the Protection of Vertebrate Animals used for Experimental and Other Scientific Purposes) and the UK Home Office Scientific Procedures Act (1986) with project approval from the University College London Institutional Animal Welfare and Ethical Review Committee.

Note that full information on the approval of the study protocol must also be provided in the manuscript.
